# Mass transfer of microbubble in liquid under multifrequency acoustic excitation - A theoretical study

**DOI:** 10.1016/j.ultsonch.2024.106760

**Published:** 2024-01-06

**Authors:** Xiong Wang, Xiao Yan, Qi Min

**Affiliations:** a Key Laboratory of Advanced Reactor Engineering and Safety of Ministry of Education, Collaborative Innovation Center of Advanced Nuclear Energy Technology, Institute of Nuclear and New Energy Technology, Beijing 100084, China; b Department of Mechanical Engineering, City University of Hong Kong, Hong Kong 999077, China; c Key Laboratory of Low-grade Energy Utilization Technologies and Systems, Chongqing University, Ministry of Education, Chongqing 400030, China; d Institute of Engineering Thermophysics, Chongqing University, Chongqing 400030, China

**Keywords:** Microbubble, Growth, Mass transfer, Acoustic, Pressure

## Abstract

Microbubble’s mass transfer under external acoustic excitation holds immense potential across various technological fields. However, the current state of acoustic technology faces limitations due to inadequate control over bubble size in liquids under external excitation. Here, we conducted numerical investigations of the mass transfer behavior of microbubbles in liquids under multifrequency acoustic excitations with different frequencies (in the MHz range), pressure amplitudes (in the range of several atmospheric pressures), and amplitude ratios. We identified various pressure threshold regions for the growth of gas bubbles (radii range from a few microns to tens of microns) and observed common intersections between single and multifrequency excitations that enable effective control of the growth intervals and final size of bubbles by adjusting the ratio of pressure amplitude and frequency value. Allocating power to the lower frequency component of multifrequency acoustic excitation is recommended to facilitate mass transfer or diffusion, as small-frequency acoustic excitation has a more significant effect than the higher frequency in the growth region. Our study provides a better understanding of the dynamics of bubbles under complex excitations and has practical implications for developing methods to control and promote bubble-related processes.

## Graphical abstract

**Figure:**
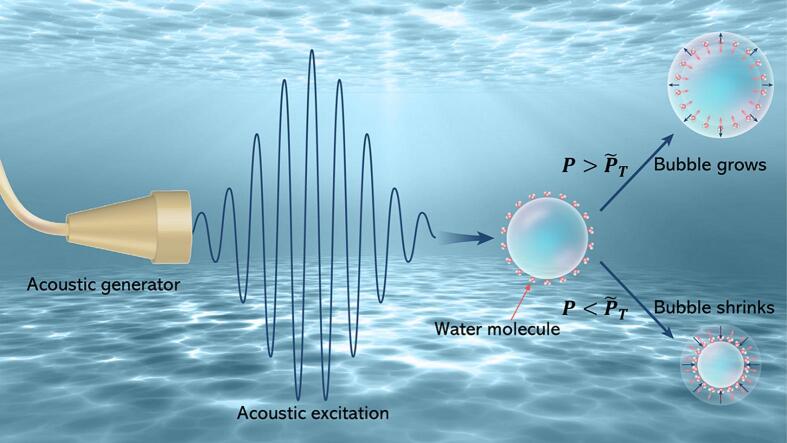

## 1 Introduction

Microbubble generation finds diverse applications across fields such as medicine, pharmacology, material science, food industry, and interdisciplinary applications like sonochemistry, sonoluminescence, and acoustic microstreaming [1], [2], [3], [4], [5], [6], [7]. Various emerging technologies have been developed to generate microbubbles in liquid, employing different physical principles such as outer liquid flow, acoustic field, and electric field [6], [8]. Among these methods, the acoustic method is one of the most critical methods due to its inherent advantages of simplicity and the ability to generate microbubbles precisely when and where they are required [9], [10], [11], [12]. These properties hold immense value across different domains, ranging from medical applications [13], [14], [15], [16] to the production of specialized chemicals, such as controlling blood clotting and detecting gastrointestinal bleeding [17], [18].

One common phenomenon in the acoustic field is rectified diffusion of gas bubbles, which refers to the process in which a pulsating gas bubble grows due to a net mass inflow when exposed to sound excitation with an amplitude greater than 0.1 MPa [19], [20], [21], [22], [23]. This mass transfer process becomes significant when a liquid containing dissolved gas is subjected to a sufficiently intense sound field. Harvey [24] first reported on this mass transfer process, and subsequent researchers have provided theoretical explanations mainly based on changes in the bubble’s surface area. Hsieh [25] extended the understanding by considering the convection term and successfully predicted mass transfer through experimental comparisons. Eller [26] proposed a theory of nonlinear bubble pulsation using a thin-diffusion layer approximation, neglecting gas diffusion and separating the diffusion equation from the bubble motion equation, an approach widely adopted by other researchers [27], [28], [29], [30], [31], [32], [33], [34], [35], [36]. Additionally, Zhang [35], [36] discussed the rectified mass diffusion of non-Newtonian fluids, such as viscoelastic fluids found in various organisms. Recently, Smith and Wang introduced an exceptional model for bubble growth in liquid through rectified diffusion [37], [38]. Their model offers a straightforward yet precise solution for gas behavior in liquid, enabling accurate predictions of bubble growth over millions of oscillation cycles [37], [38]. Notably, their findings highlight the significant influence of shell and area effects on bubble growth in liquid with bulk surfactant concentrations below 2.4 mM, underscoring the importance of surface tension in rectified diffusion for aqueous surfactant solutions [39]. For further theoretical studies on this mass transfer process, readers are recommended to refer to Fyrillas’s work [33], [34], [40].

Significant advancements in theoretical research over the past few decades have led to the development of various acoustic devices for applications in medicine and chemistry [4], [6], [23], [41]. However, these devices face a common and challenging limitation: it is challenging to generate controllable-sized microbubbles in fluid, which is crucial for the field of medical imaging, biomedical, environmental, and chemical reactions. For example, in green biorefinery, precise control over bubble size is desired for targeted extraction of bioactive compounds [42]. Furthermore, the use of contrast agents in microbubbles for treating blood–brain barrier disruption is hindered by limitations in the controllable bubble size [43]. These fields all require quantitative control of bubble size in liquids to meet specific application demands.

Recent studies have shown promising results by employing dual-frequency external acoustic excitation, effectively dividing the range of bubble growth into two smaller ranges [22], [35], [21], [44], [45], [46]. In addition, dual-frequency sonication can effectively suppress chaotic bubble oscillations [47] and reduce the threshold for inertial cavitation, thereby enhancing power efficiency [48]. Considering the vast number of parameter combinations involved [49], [50], researchers have developed GPU-based methods to investigate the dynamics of multi-frequency bubbles. These findings collectively underscore the advantages of employing multi-frequency acoustic excitation in diverse applications and offer a pathway for optimizing ultrasound stimulation to induce inertial cavitation. Both works reaffirm the benefit of using multi-frequency acoustic excitation for various applications and provide a route for optimizing ultrasound excitations for initiating inertial cavitation. Therefore, exploring the increase in the number of acoustic frequencies emerges as an intelligent and highly promising approach to achieving directed and precise control over bubble size in liquid systems. However, the specific theory and method for achieving rational control of bubble sizes through modulation of parameters in the external acoustic field remain unknown, impeding the development of strategies aiming to control the bubble’s mass transfer process.

To overcome this limitation, our work contributes to understanding the mass transfer process under multifrequency acoustic excitation, primarily focusing on the theoretical method. Through theoretical and numerical calculations, we investigated how three critical parameters—frequency, pressure amplitude, and amplitude ratio—affect the growth and behavior of microbubbles in liquid under acoustic excitations. By uncovering the underlying mechanisms behind the mass transfer phenomenon, our study provides valuable insights into effectively utilizing multifrequency acoustic stimulation for precise control and enhancement of bubble-related processes. The implications of our findings extend to various fields, including medicine, pharmacology, material science, and the food industry.

## 2 Theoretical method

To derive the theoretical equations, we began by assuming that the external acoustic excitations, denoted as $P_{s}\left(t\right)$, could be expressed as a multifrequency acoustic signal consisting of three frequencies with varying amplitudes

(1) $$P_{s}\left(t\right)=P_{0}+P_{A_{1}}\cos\left(\omega_{1}t\right)+P_{A_{2}}\cos\left(\omega_{2}t\right)+P_{A_{3}}cos\left(\omega_{3}t\right)$$

**Fig. 1:**
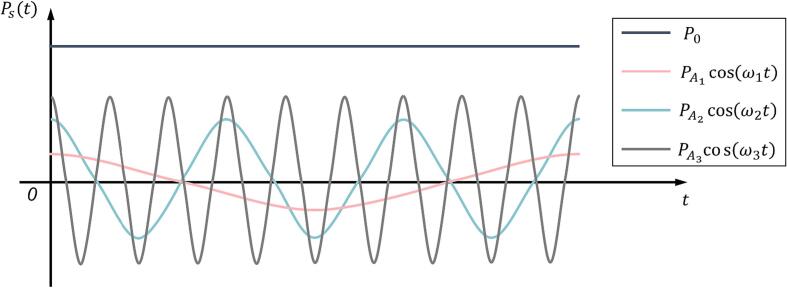
Schematic diagram of multifrequency acoustic excitation, with the assumption that $P_{A_{1}}<P_{A_{2}}<P_{A_{3}}<P_{0}$ in this illustration.

Here, $P_{0}$ represents the ambient pressure and $P_{A_{i}}$ (i=1,2,3) represents the amplitudes of each external acoustic excitation with angular frequencies of $\omega_{1}$, $\omega_{2}$ and $\omega_{3}$, respectively. The schematic diagram of the external acoustic excitations is shown in Fig. 1.

Assuming the working fluid is a Newtonian fluid, we adopt Keller’s equation [51] to take into consideration the liquid’s compressibility and viscosity. The bubble motion equation is

(2) $$\left(1-\frac{\dot{R}}{c_{l}}\right)R\ddot{R}+\frac{3}{2}\left(1-\frac{\dot{R}}{3c_{l}}\right){\dot{R}}^{2}=\left(1+\frac{\dot{R}}{c_{l}}\right)\frac{p_{\mathrm{ext}}\left(R,t\right)-p_{s}\left(t\right)}{\rho_{l}}+\frac{R}{\rho_{l}c_{l}}\frac{d\left[p_{\mathrm{ext}}\left(R,t\right)-p_{s}\left(t\right)\right]}{\mathrm{dt}}$$

where

(3) $$p_{\mathrm{ext}}\left(R,t\right)=P_{\mathrm{in}}-\frac{2\sigma }{R}-\frac{4\mu_{l}}{R}\dot{R}$$

(4) $$P_{\mathrm{in}}=\left(P_{0}+\frac{2\sigma }{R_{0}}\right){\left(\frac{R_{0}}{R}\right)}^{3/\kappa }$$

Here, the over dot is the time derivative, R is the instantaneous bubble radius, $c_{l}$ is the sound speed in liquid, $\rho_{l}$ is the liquid density, t is time, $P_{\mathrm{in}}$ is the instantaneous pressure at the gas side, $R_{0}$ is the equilibrium bubble radius, σ is the surface tension, κ is the polytropic exponent, $\mu_{l}$ is the liquid viscosity. The acoustic field has three different frequencies, which means the acoustic field with triple frequencies. It should be noted that our model does not consider variations in surface tension, which limits its applicability to non-Newtonian fluids and the dynamics of coated bubbles in liquid [39]. Additionally, our model only incorporates the linear approximation of bubble oscillation and does not account for highly nonlinear phenomena, including sub-harmonics and bifurcation [52], [53], [54]. Moreover, we assume that the bubble’s amplitude is small, resulting in oscillations within the spherical regime [55], [56], [57].

The diffusion equation follows Fick’s law and considers the gas that is dissolved in the liquid. The gas concentration c in liquid can be written as

(5) $$\frac{\partial c}{\partial t}+u∙\nabla c=D\nabla^{2}c$$

where u is the velocity of the liquid at one point; D is the diffusion constant. Considering the initial and boundary conditions [58],

(6) $$c\left(r,0\right)=C_{i},r>R$$

(7) $$\lim_{r\to \infty }c\left(r,t\right)=C_{i}$$

(8) $$c\left(R,t\right)=C_{s},t>0$$

Here, $C_{i}$ is the gas’s initial concentration and $C_{s}$ is the gas concentration in the liquid. $C_{s}$ is controlled by Henry’s law, which suggests that it is directly proportional to the partial pressure of the gas. Specifically, $C_{0}={k_{H}}^{-1}P_{0}$ and $C_{s}=k_{H}^{-1}\left(P_{0}+2\sigma /R\right)$, where $C_{0}$ is the saturation concentration, $k_{H}$ is the Henry constant. The first term of Eq. (5), i.e., the convective term, represents the transient change of concentration of the gas. We can neglect it due to the slow movement and the diffusion equation can be simplified as $u\cdot \nabla c=D\nabla^{2}c$. Hence, the bubble motion and mass transfer of Eq. (5) could be uncoupled. Combining Eq. (1) to Eq. (8), we can obtain the bubble growth rate. It can be expressed as [1], [3], [31]

(9) $$\frac{dR_{0}}{\mathrm{dt}}=\frac{DR_{g}T_{\infty }C_{0}}{R_{0}P_{0}}\left[\langle R/R_{0}\rangle +R_{0}{\left(\frac{{\left(R/R_{0}\right)}^{4}}{\pi tD}\right)}^{1/2}\right]\times {\left(1+\frac{4\sigma }{3P_{0}R_{0}}\right)}^{-1}\left(\frac{C_{i}}{C_{0}}-\frac{{\left(R/R_{0}\right)}^{4}\left(P_{\mathrm{in}}/P_{0}\right)}{{\left(R/R_{0}\right)}^{4}}\right)$$

Here, $R_{g}$ is the universal gas constant, $T_{\infty }$ is the ambient temperature in the liquid, represents the time average. The solution of Eq. (9) is

(10) $$\frac{R}{R_{0}}=1+B_{2}{\left(P_{A_{1}}/P_{0}\right)}^{2}+\left[\sum_{i=1}^{3}A_{1i}cos\left(\omega_{i}t+\delta_{1i}\right)\right]\left(P_{A_{1}}/P_{0}\right)$$

and

(11) $$A_{1i}=-\frac{P_{0}P_{A_{i}}}{M\rho_{l}R_{0}^{2}P_{A_{1}}}{\left[\frac{1+{\left(\omega_{i}R_{0}/c_{l}\right)}^{2}}{{\left(\omega_{0}^{2}-\omega_{i}^{2}\right)}^{2}+4\beta_{\mathrm{tot}}^{2}\omega_{i}^{2}}\right]}^{1/2}$$

(12) $$\delta_{1i}={tan}^{-1}\left\{\frac{\left(\omega_{i}R_{0}/c_{l}\right)\left(\omega_{0}^{2}-\omega_{i}^{2}\right)-2\beta_{\text{tot}\;}\omega_{i}}{\left(\omega_{0}^{2}-\omega_{i}^{2}\right)+2\beta_{\mathrm{tot}}\omega_{i}^{2}R_{0}/c_{l}}\right\}$$

(13) $$B_{2}=-\frac{1}{4\omega_{0}^{2}M}\sum_{i=1}^{3}A_{1i}^{2}\omega_{i}^{2}+\frac{1}{4}\frac{\sum_{i=1}^{3}A_{1i}^{2}}{\rho_{l}R_{0}^{2}\omega_{0}^{2}M}\left[3\kappa \left(3\kappa +1\right)\left(P_{0}+\frac{2\sigma }{R_{0}}\right)-\frac{4\sigma }{R_{0}}\right]$$

where i=1,2,3, and $\omega_{0}^{2}$, $\beta_{\mathrm{tot}}$ and M are

(14) $$\omega_{0}^{2}=\frac{1}{\rho_{l}R_{0}^{2}M}\left[3\kappa \left(P_{0}+\frac{2\sigma }{R_{0}}\right)-\frac{2\sigma }{R_{0}}\right]$$

(15) $$\beta_{\mathrm{tot}}=\frac{2\mu_{l}}{\rho_{l}R_{0}^{2}M}+\frac{R_{0}}{2c_{l}}\omega_{0}^{2}$$

(16) $$M=1+\frac{4\mu_{l}}{\rho_{l}R_{0}^{2}}\frac{R_{0}}{c_{l}}$$

Here, we ignore initial conditions and the solution of the homogeneous equation’s effect on gas bubble motion. The time averages of $P_{A_{1}}/P_{0}$ are determined (considering solutions up to the second order) as

(17) $$\langle R/R_{0}\rangle =1+B_{2}{\left(P_{A_{1}}/P_{0}\right)}^{2}$$

(18) $$\langle {\left(R/R_{0}\right)}^{4}\rangle =1+\left[4B_{2}+3\left(A_{11}^{2}+A_{12}^{2}+A_{13}^{2}\right)\right]{\left(P_{A_{1}}/P_{0}\right)}^{2}$$

(19) $$\langle {\left(R/R_{0}\right)}^{4}\left(P_{\mathrm{in}}/P_{0}\right)\rangle =\left(1+\frac{2\sigma }{P_{0}R_{0}}\right)\left\{1+\left[\left(4-3\kappa \right)B_{2}+\frac{\left(4-3\kappa )(3-3\kappa \right)}{4}\sum_{i=1}^{3}A_{1i}^{2}\right]{\left(P_{A_{1}}/P_{0}\right)}^{2}\right\}$$

By combining Eqs. (17)–(19) and Eq. (9), we can obtain the bubble growth rate. To establish a clear relationship between the pressure amplitudes of the acoustic excitation with three different frequencies and the threshold of acoustic pressure amplitude of mass diffusion ${\tilde{P}}_{T}$, we assume that $P_{A_{1}}=P_{A_{2}}=P_{A_{3}}$. Therefore, combining with Eqs. (5)–(7) and setting $dR_{0}/dt=0$, we can obtain

(20) $${{\tilde{P}}_{T}}^{2}=\frac{\left(1+\frac{2\sigma }{R_{0}P_{0}}\right)-C_{i}/C_{0}}{\frac{\sum_{i=1}^{3}A_{1i}^{2}}{P_{0}^{2}}\left[\frac{3C_{i}}{C_{0}}-\frac{3(4-3\kappa )(1-\kappa )}{4}\left(1+\frac{2\sigma }{R_{0}P_{0}}\right)\right]+\frac{B_{2}}{P_{0}^{2}}\left[\frac{4C_{i}}{C_{0}}-\left(1+\frac{2\sigma }{R_{0}P_{0}}\right)\left(4-3\kappa \right)\right]}$$

**Fig. 2:**
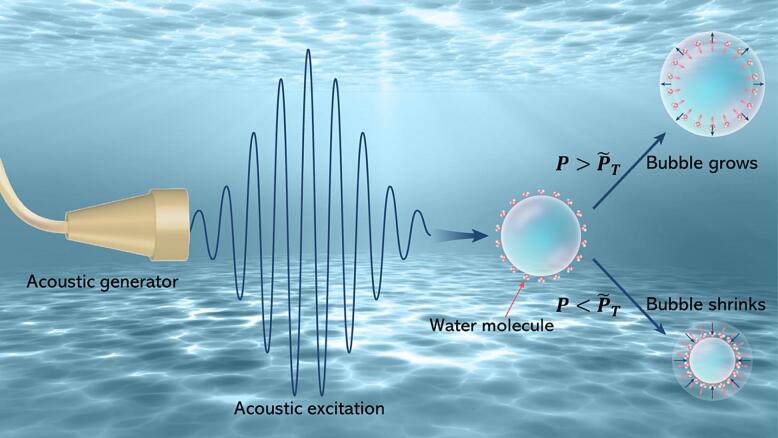
The schematic diagram illustrates the dynamic behavior of a bubble in liquid under acoustic excitations, depicting its growth (with water molecules moving from the liquid through the bubble interface to the inside of the bubble) or shrinkage (with water molecules moving from inside the bubble through the bubble interface to the liquid).

Therefore, as shown in Fig. 2, if the acoustic pressure amplitude exceeds the threshold value of ${\tilde{P}}_{T}$, the bubble will grow gradually. However, if the acoustic pressure amplitude is lower than ${\tilde{P}}_{T}$, the bubble will shrink and eventually collapse.

## 3 Results and discussions

The frequency values considered in our discussion are in the megahertz range, which is commonly used in various fields, such as the scattering cross-section of acoustic bubbles and ultrasonic wave propagation [4], [6]. Specifically, we considered two different ratios: $\omega_{1}:\omega_{2}:\omega_{3}=1:2:3$ and 1:3:9, with $\omega_{1}=5\times 10^{5}$/s. To quantify the pressure amplitudes, we defined $N_{i}$ as the ratio of pressure amplitude, where $N_{1}$ as the ratio of pressure amplitude $P_{A2}$ to $P_{A1}$, and $N_{2}$ as the ratio of pressure amplitude $P_{A3}$ to $P_{A1}$. Therefore, the pressure amplitudes have a ratio of $P_{A1}:P_{A2}:P_{A3}=1:N_{1}:N_{2}$, and the total input pressure (equivalent pressure) is given by $P_{e}={\left({P_{A1}}^{2}+{P_{A2}}^{2}+{P_{A3}}^{2}\right)}^{1/2}$. To maintain a constant value for ${\left(1+N_{1}+N_{2}\right)}^{2}$ throughout our discussion, we keep $P_{e}$ constant for different values of $N_{1}$ and $N_{2}$. This means that the total input power remains constant. We consider the case of air bubbles in water, and the constants used in our numerical calculation are $P_{0}=1.01\times 10^{5}$ Pa, $\rho_{l}=998$ kg/m^3^, $c_{l}=1486$ m/s, $\mu_{l}=0.001$ Pa·s, σ=72.8 mN/m, $D=2.4\times 10^{-9}$ m^2^/s, $R_{g}=8.314$ J/mol/K, $T_{\infty }=293.15$ K, κ=1.33.

### 3.1 Bubble growth region

To begin, we investigate how the amplitude and frequency of multifrequency acoustic excitation impact the mass transfer process. To simplify our analysis, we assume that the amplitudes of all frequencies in the multifrequency excitation are equal to $P_{0}$, and that the total power of the excitation $P_{e}=\sqrt{3}P_{0}$ remains constant for different values of $N_{1}$ and $N_{2}$. Accordingly, the pressure amplitudes of the three acoustic excitations are given by: $P_{A1}=P_{0}\sqrt{3/(1+N_{1}^{2}+N_{2}^{2})}$; $P_{A2}=P_{0}\sqrt{3N_{1}^{2}/\left(1+N_{1}^{2}+N_{2}^{2}\right)}$; $P_{A3}=P_{0}\sqrt{3N_{2}^{2}/\left(1+N_{1}^{2}+N_{2}^{2}\right)}$. The total threshold pressure amplitude and the pressure amplitudes of three acoustic excitations are denoted as $P_{\mathrm{Te}}$, $P_{TA1}$, $P_{TA2}$, $P_{TA3}$, respectively, and are related by the equation $P_{\mathrm{Te}}={\left({P_{TA1}}^{2}+{P_{TA2}}^{2}+{P_{TA3}}^{2}\right)}^{1/2}$. Therefore, $P_{e}>P_{\mathrm{Te}}$ indicates that the bubble will grow until when $P_{e}=P_{\mathrm{Te}}$.

**Fig. 3:**
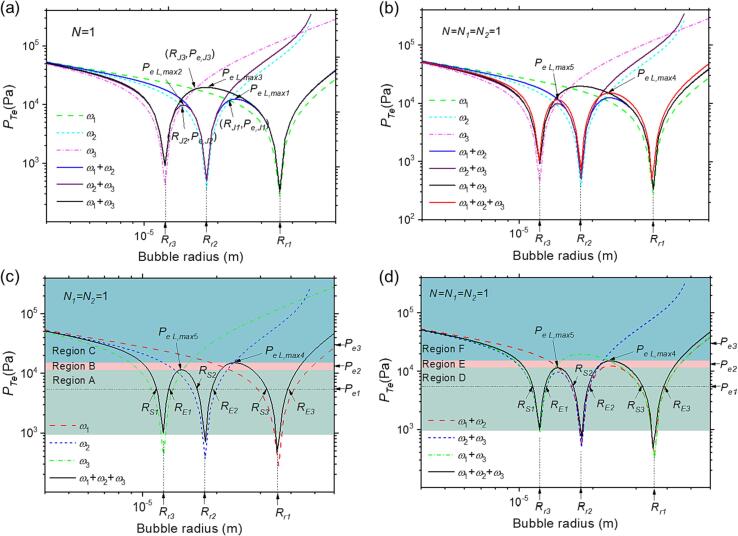
The acoustic pressure amplitude thresholds required to transfer gas bubble mass in liquid under different acoustic excitation conditions. (a) single and dual frequencies, (b) single, dual, and multifrequency, (c) single and multifrequency, (d) dual and multifrequency. Here, the excitation frequencies, $\omega_{1}=5\times 10^{5}$ /s, $\omega_{2}=1\times 10^{6}$ /s, $\omega_{3}=1.5\times 10^{6}$ /s, and $N=N_{1}=N_{2}=1$, where $N=P_{A1}/P_{A2}$ for dual-frequency acoustic excitation. $R_{r1}$, $R_{r2}$ and $R_{r3}$ represent the resonance bubble radius of gas bubbles. $\left(R_{J1},P_{J1}\right)$, $\left(R_{J2},P_{J2}\right)$ and $\left(R_{J3},P_{J3}\right)$ represent the intersection points of all the threshold curves while $P_{\mathrm{eL},max1}$, $P_{\mathrm{eL},max2}$ and $P_{\mathrm{eL},max3}$ represent the local maximum of threshold values of dual frequencies. $R_{\mathrm{Si}}$, $R_{\mathrm{Ei}}$, (the subscript i=1,2,3) denote the start and end radius of bubble growth regions, respectively. $P_{\mathrm{eL},max4}$, $P_{\mathrm{eL},max5}$ represent the local maximum of threshold values of triple frequencies. $P_{\mathrm{ei}}$ (the subscript i=1,2,3) represent different values in (c) and (d). Regions of A B C in (c) and D E F in (d) are signed with different colors.

To verify the accuracy of our theory, we compare the changes in pressure threshold and corresponding first-order frequency (i.e., $N_{1}$ and $N_{2}$ equal to zero) threshold of our model at the second-order frequency (either $N_{1}$ or $N_{2}$ equal to zero), as shown in Fig. 3a. We observed that the threshold pressure values ($P_{\mathrm{Te}}$) under single and corresponding dual-frequency acoustic excitations intersected at one point on the value line, and the maximum pressure threshold value was observed for the dual-frequency acoustic excitations. For example, the value of $P_{\mathrm{Te}}$ under single frequency ($\omega_{1}$ or $\omega_{2}$) and dual frequencies acoustic excitations ($\omega_{1}+\omega_{2}$) intersected at the common point $\left(R_{J1},P_{e,J1}\right)$, which is consistent with the previous research [46]. However, unlike the situation where they share the same intersection points, the value of $P_{\mathrm{Te}}$ under multifrequency excitation did not intersect on the common intersect points with both single and dual frequencies (Fig. 3b, red line). Furthermore, its threshold pressure was higher than both single and dual-frequency. We distinguished the maximum value of the dual-frequency, i.e., $P_{\mathrm{eL},max1}$, $P_{\mathrm{eL},max2}$ and $P_{\mathrm{eL},max3}$ in Fig. 3a, with the maximum of the triple frequency, $P_{\mathrm{eL},max4}$ and $P_{\mathrm{eL},max5}$ in Fig. 3b.

We then compare the pressure threshold of single and triple frequencies in Fig. 3c, which shows three regions (A, B, and C) of different $P_{\mathrm{Te}}$ based on the value of $P_{e\;L,max4}$ and $P_{e\;L,max5}$ to enable a clear comparison between these lines. A dotted line labeled as $P_{\mathrm{ei}}$ is defined, which intersects with $P_{\mathrm{Te}}$ at the points $R_{\mathrm{Si}}$ and $R_{\mathrm{Ei}}$, where the subscript i=1,2,3, represents bubbles under acoustic excitations in the region $\left(R_{S},\;R_{E}\right)$ can grow from $R_{S}$ to its final size $R_{E}$. In region A, where ${P_{e1}<min(P}_{e\;L,max4},\;P_{e\;L,max5})$, we observe six intersections between the threshold curve and $P_{e}$. We find that, under the same total input power, the bubble growth region under multifrequency acoustic excitation does not significantly increase compared to single-frequency acoustic excitations. Although increasing the frequency can increase the number of intervals in which the bubble grows, the corresponding narrowing of the bubble growth interval at the corresponding frequency means that the growth region under excitation remains nearly unchanged. In region B, where $P_{e2}\in \left(P_{\mathrm{eL},max5},\;P_{\mathrm{eL},max4}\right)$, bubbles with radii in the region $\left(R_{S1},R_{E2}\right)$ can grow under multifrequency acoustic excitation from $R_{S1}$ to the final equilibrium bubble radius $R_{E2}$. Furthermore, in region C, where ${P_{e3}>max(P}_{e\;L,max4},\;P_{e\;L,max5})$, we note that bubbles with radii in the region $\left(R_{S1},R_{E2}\right)$ can both grow to $R_{E2}$, indicating a significant increase compared to the single frequency $\omega_{2}$ or $\omega_{3}$. In other words, adding more low-frequency acoustic excitation is beneficial for increasing the bubble growth region.

Next, we compare the pressure threshold of dual and multifrequency in Fig. 3d, where different regions can also be distinguished using $P_{\mathrm{eL},max4}$ and $P_{\mathrm{eL},max5}$. In region D, where ${P_{e1}<min(P}_{e\;L,max4},\;P_{e\;L,max5})$, the bubble growth regions under multifrequency acoustic excitation remain almost the same as those under dual-frequency acoustic excitations, similar to region A. In region E, where $P_{e2}\in \left(P_{\mathrm{eL},max4},\;P_{\mathrm{eL},max5}\right)$), four intersections exist between the threshold curve and $P_{e2}$. Bubbles with radii in the region $\left(R_{S1},R_{E2}\right)$ can grow to the final equilibrium bubble radius $R_{E2}$. Moreover, the local maximum threshold values of dual-frequency $P_{\mathrm{eL},max1}$, $P_{\mathrm{eL},max2}$ and $P_{\mathrm{eL},max3}$ will influence the growth region to a limited extent. In region F, where ${P_{e3}>max(P}_{e\;L,max4},\;P_{e\;L,max5})$, two intersections exist between the threshold curve and $P_{e3}$. The situation is similar to that in region C, where the dual frequencies contain the single frequency $\omega_{1}$, and the growth region increases significantly under triple-frequency acoustic excitations. Therefore, similar to dual-frequency acoustic excitation, multifrequency acoustic excitation can also expand the microbubble’s growth region by adding an additional low-frequency acoustic excitation.

### 3.2 Influence of $N_{1}$ and$N_{2}$

**Fig. 4:**
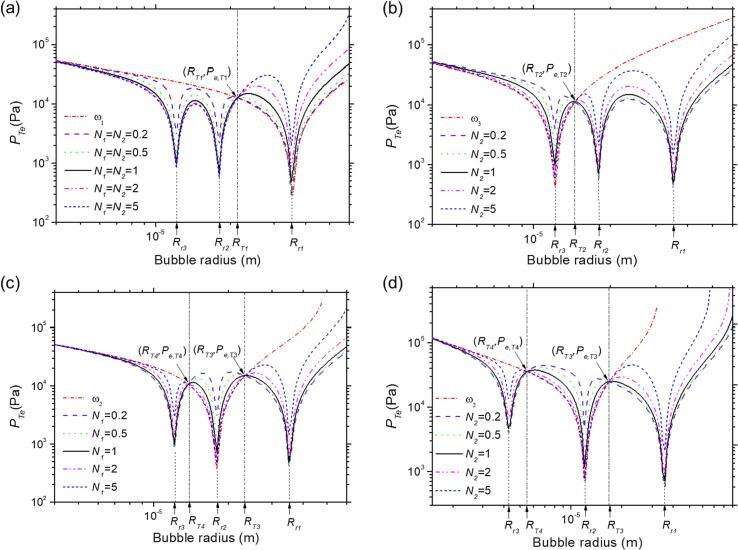
The predicted threshold of the total acoustic pressure amplitude of gas bubble mass transfer in liquid under acoustic excitation conditions with multifrequency [$\omega_{1}+\omega_{2}+\omega_{2}$] and different single frequencies: (a) $\omega_{1}$, (b) $\omega_{3}$, (c) $\omega_{2}$, with $\omega_{1}=5\times 10^{5}$ /s, $\omega_{2}=1\times 10^{6}$ /s, $\omega_{3}=1.5\times 10^{6}$ /s^1^. (d) $\omega_{3}$ with $\omega_{1}=5\times 10^{5}$ /s, $\omega_{2}=1.5\times 10^{6}$ /s, $\omega_{3}=4.5\times 10^{6}$ /s. $N_{1}=N_{2}=0.2,0.5,1,2,5$, respectively. The intersection points of all the threshold curves are denoted as $\left(R_{\mathrm{Ti}},P_{e,Ti}\right)$, where the subscript i=1,2,3,4.

Although the above discussion highlights the complex nature of bubble growth under multifrequency acoustic excitation during mass transfer processes, we can also discover some universal laws that further clarify this problem. Interestingly, as depicted in Fig. 4, the predicted threshold value of $P_{\mathrm{Te}}$ does have common intersect points under single and triple frequencies with different $N_{1}$ and $N_{2}$ values. Here, we identify one or two fixed points for all conditions, denoted as $\left(R_{T1},P_{e,T1}\right)$ $\left(R_{T2},P_{e,T2}\right)$ $\left(R_{T3},P_{e,T3}\right)$ and $\left(R_{T4},P_{e,T4}\right)$, respectively. By changing the value of $N_{1}$ and $N_{2}$, these points regulate the local value of $P_{\mathrm{eL},max}$, thereby influencing the bubble growth region as emphasized above. In Fig. 4a, when $N_{1}=N_{2}$ (i.e. $P_{A1}=P_{A2}=P_{A3}$), the $P_{\mathrm{Te}}$ lines under single-frequency ($\omega_{1}$) and multifrequency (${\omega_{1}+\omega }_{2}+\omega_{3}$) acoustic excitation intersect at the point $\left(R_{T1},P_{e,T1}\right)$, regardless of how $N_{1}$ and $N_{2}$ change. Similarly, in Fig. 4b, when $N_{2}$ changes and $N_{1}$ remains fixed, the $P_{\mathrm{Te}}$ lines under single frequency ($\omega_{3}$) and multifrequency acoustic excitation intersect at the point $\left(R_{T2},P_{e,T2}\right)$, regardless of how $N_{2}$ changes. Likewise, in Fig. 4c, when $N_{1}$ changes and $N_{2}$ remains fixed, the $P_{\mathrm{Te}}$ lines under single-frequency ($\omega_{2}$) and multifrequency acoustic excitation intersect at the point $\left(R_{T3},P_{e,T3}\right)$ and $\left(R_{T4},P_{e,T4}\right)$, respectively, independent of the value of $N_{1}$. Appendix A demonstrates these intersection points ($\left(R_{\mathrm{Ti}},P_{e,Ti}\right)$, where i=1,2,3,4) under three different conditions from the perspective of theoretical models. These intersection points indicate that the threshold of the mass diffusion under acoustic excitation conditions with multifrequency is independent on the $N_{1}$ and $N_{2}$. When the multifrequency value ratio ($\omega_{1}:\omega_{2}:\omega_{3}$) changes from 1:2:3 to 1:3:9, as shown in Fig. 4d, we find that the $P_{\mathrm{Te}}$ lines under single and multifrequency under different $N_{1}$ also intersect at two points $\left(R_{T3},P_{e,T3}\right)$ and $\left(R_{T4},P_{e,T4}\right)$, respectively. Despite the complexity, we still demonstrate that there are fixed intersection points of $P_{\mathrm{Te}}$ lines between the multifrequency and the single-frequency acoustic excitation.

Furthermore, we note in Fig. 4a that when $N_{1}=N_{2}<1$, increasing $N_{1}$ and $N_{2}$ makes the threshold curves near the resonance bubble radius much narrower on the right region of the curve, while those on the left region remain almost the same as the one under single-frequency excitation with frequency. In Fig. 4b, when $N_{2}<1$ varies and $N_{1}$ remain the same, decreasing $N_{2}$ makes the threshold curves near the resonance bubble radius much narrower on the left region of the curve, while those on the right region remain almost the same as the one under single-frequency excitation. However, when $N_{1}$ is less than 1 in Fig. 4c, decreasing $N_{1}$ makes the threshold curves near the resonance bubble radius much narrower in the middle region $\left(R_{T4},R_{T3}\right)$ of the curve, while the outside of the region almost remains the same as the one under single-frequency excitation. Therefore, these results demonstrate that the presence of common intersection points, a fascinating phenomenon similar to the fixed wave nodes observed in mechanical, electromagnetic, or other types of waves [59], will affect the region where bubble growth occurs. Therefore, we can regulate the bubble growth region by controlling the value of $N_{i}$ through our demands.

**Fig. 5:**
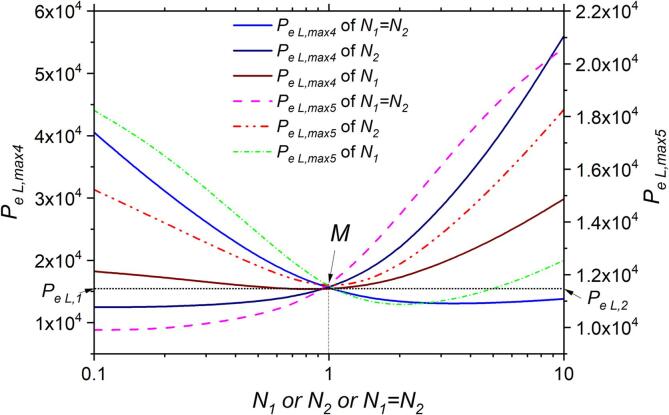
Predicted local maximum threshold pressure (and) against the ratio of two excitation acoustic pressure amplitudes ($N_{1}$, $N_{2}$ or $N_{1}=N_{2}$ respectively). $\omega_{1}=5\times 10^{5}$ /s, $\omega_{2}=1.5\times 10^{6}$ /s, $\omega_{3}=4.5\times 10^{6}$ /s.

To further clarify how to regulate $P_{\mathrm{eL},max}$ and bubble growth region through $N_{i}$ values, we investigated the impact of the pressure ratio ($N_{1}$ and $N_{2}$) on the local maximum threshold pressure ($P_{e\;L,max4}$, $P_{e\;L,max5}$) of multifrequency excitation. Fig. 5 shows that $N_{1}$ and $N_{2}$ have a significant effect on the value of $P_{e\;L,max4}$ and $P_{\mathrm{eL},max5}$, and they both cross at a point M where $N_{1}=N_{2}=1$, $P_{\mathrm{eL},1}=15500$ Pa and $P_{\mathrm{eL},2}=11500$ Pa, indicating the best value to enhance bubble growth is $N_{1}=N_{2}=1$. In addition, we summarized the bubble growth regions under different acoustic excitations with different values of $N_{1}$, $N_{2}$ in Table 1. We compared the bubble growth regions under different N values in the range of $P_{e}{\in (P}_{e\;L,max4,N=1},\;P_{e\;L,max5,N=5})$ under single and multifrequency excitation. We found that when the N value is less than 1 (e.g., N=0.2), the wide of bubble growth region (43.85 μm) under multifrequency excitation is slightly larger than that under single-frequency excitation (40.81 μm). By adding two high-frequency acoustic excitations ($\omega_{2}=1\times 10^{6}\;/s$ and $\omega_{3}=1.5\times 10^{6}\;/s$) to the single-frequency excitation ($\omega_{1}=5\times 10^{5}\;/s$), the bubble growth region can increase from approximately 40 μm to 44 μm, representing a 10 % increase. However, by adding two low-frequency acoustic excitations ($\omega_{1}=5\times 10^{5}\;/s$, $\omega_{2}=1\times 10^{6}\;/s$) to the single-frequency excitation ($\omega_{3}=1.5\times 10^{6}\;/s$), the bubble growth region can increase from approximately 8.7 μm to 44 μm, representing a four-fold increase. However, this can cause a decrease in droplet size (from 7.82 μm to a minimum of approximately 8.48 μm, a decrease of approximately 8 %) at the onset of bubble growth. Therefore, it is necessary to appropriately adjust the *N* value and the magnitude of different frequencies based on the specific application scenario to control the mass transfer process of bubbles effectively.

**Table 1:** Bubble growth regions of different approaches and $N_{1}$, $N_{2}$. $N_{1}$, $N_{2}$ and $N_{1}=N_{2}$ equals 0.2, 0.5, 1, 2, 5, respectively. $\omega_{1}=5\times 10^{5}\;/s$, $\omega_{2}=1\times 10^{6}\;/s$, $\omega_{3}=1.5\times 10^{6}\;/s$.

*P*_e_ (kPa)	Number of frequency types	*N*_1_ or *N*_2_	Bubble growth region (um) (*R*_E_, *R*_S_)	Region Wide
*P*_e_ = 60	Triple frequencies *P*_e_∈(*P*_e L,max4,N=1_, *P*_e L,max4,N=5_)	*N*_1_ = 0.2	(8.70, 49.85)	41.15
		1	(8.95, 47.27)	38.32
		5	(9.91, 25.62); (30.01, 39.78)	25.48
		*N*_2_ = 0.2	(10.52, 50.00)	39.48
		1	(8.95, 47.27)	38.32
		5	(7.96, 19.75); (30.74, 39.58)	20.63
		*N*_1_ = *N*_2_ = 0.2	(10.75, 54.60)	43.85
		1	(8.95, 47.27)	38.32
		5	(8.48, 23.45); (31.89, 38.50)	21.58
	Single frequency	w_1_	(14.28, 55.09)	40.81
		w_2_	(10.18, 25.29)	15.11
		w_3_	(7.82, 16.58)	8.76

### 3.3 Influence of initial concentration

**Fig. 6:**
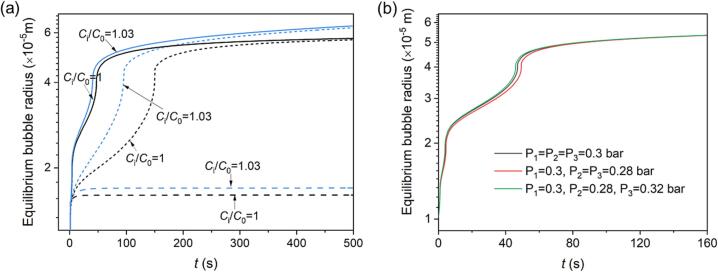
Influence of (a) initial uniform concentration and (b) aptitude of $P_{i}$ on equilibrium bubble radius. $\omega_{1}$ (dash line), $\omega_{1}+\omega_{2}$ (short dash line), $\omega_{1}+\omega_{2}+\omega_{3}$ (solid line) in (a).$\omega_{1}$ = 5*10^5^ s^−1^, $\omega_{2}$=1.5*10^6^ s^−1^, $\omega_{3}$=1*10^6^ s^−1^.

Fig. 6 illustrates the impact of the initial uniform concentration (Fig. 6a) and pressure amplitude of frequency division (Fig. 6b) on the equilibrium bubble radius. It is observed that the growth rate of bubbles under multifrequency acoustic excitation is significantly higher than that under single and dual-frequency acoustic excitation. However, the final equilibrium bubble radius of dual and triple frequencies remains unchanged. Therefore, exceeding the saturation conditions (e.g., $C_{i}/C_{0}=1.03$ in Fig. 6a) accelerates the growth rate of bubbles while maintaining the same final equilibrium bubble size. This suggests that increasing the frequency from dual to triple does not significantly affect the final size of bubbles. In addition, Fig. 6b presents cases of multifrequency acoustic excitation with unequal amplitudes. It is observed that the bubble growth rate increases as the amplitude of acoustic fields increases, as demonstrated by the difference between the red, green, and black solid lines.

## 4 Conclusions

In conclusion, we demonstrate that multifrequency acoustic excitation can enhance the mass transfer of gas bubbles in liquids. We reveal that multifrequency acoustic excitation can significantly accelerate the mass transfer process of air bubbles in liquids when its pressure exceeds a certain threshold, which is lower than that of dual-frequency acoustic excitation. Furthermore, the introduction of more frequency excitations complicates the bubble growth process and increases the number of discrete growth intervals. We identified common intersection points between triple-frequency and single-frequency acoustic excitations under equal energy input. This discovery allows for the effective control of bubble growth intervals and size by strategically adjusting the amplitude ratio parameter $N_{i}$. By increasing the number of frequencies in the external acoustic field and rationally controlling parameters such as the relative ratios between frequencies and the amplitudes of the acoustic fields, we can generate multiple bubbles of varying sizes. Such controlling size of the growth bubble in liquids by multifrequency acoustic excitation has significant implications for the field of biomedical, environmental, and chemical reaction. In the future, the nonlinear oscillations, the shell effect of vapor bubbles, and the bubble oscillations in non-Newtonian fluids should be studied to reveal the complex nature of the nonlinear properties.

## CRediT authorship contribution statement

**Xiong Wang:** Conceptualization, Data curation, Formal analysis, Investigation, Methodology, Project administration, Resources, Software, Supervision, Validation, Visualization, Writing – original draft, Writing – review & editing. **Xiao Yan:** Investigation, Writing – original draft. **Qi Min:** Funding acquisition, Supervision, Writing – review & editing.

## Declaration of competing interest

The authors declare that they have no known competing financial interests or personal relationships that could have appeared to influence the work reported in this paper.

## Data Availability

Data will be made available on request.

## Appendix A . Intersection points

Regarding the mass diffusion excited by the triple frequencies approach, the equation of threshold is

(A1) $$\frac{C_{i}}{C_{0}}-\frac{\langle {\left(R/R_{0}\right)}^{4}\left(P_{\mathrm{in}}/P_{0}\right)\rangle }{\langle {\left(R/R_{0}\right)}^{4}\rangle }=0$$

Using Eqs. (17)–(19), Eq. (A1) can be expressed as,

(A2) $$K_{0}=K_{1}\cdot A_{1}^{2}\cdot \left(P_{A1}^{2}/P_{0}^{2}\right)+K_{2}\cdot A_{2}^{2}\cdot \left(P_{A2}^{2}/P_{0}^{2}\right)+K_{3}\cdot A_{3}^{2}\cdot \left({P_{A3}}^{2}/P_{0}^{2}\right)$$

where

$$A_{1}=-\frac{P_{0}}{M\rho_{l}R_{J}^{2}}{\left[\frac{1+{\left(\omega_{1}R_{J}/c_{1}\right)}^{2}}{{\left(\omega_{0}^{2}-\omega_{1}^{2}\right)}^{2}+4{\beta_{\text{tot}}}^{2}\omega_{1}^{2}}\right]}^{1/2}$$

$$A_{2}=-\frac{P_{0}}{M\rho_{l}R_{J}^{2}}{\left[\frac{1+{\left(\omega_{2}R_{J}/c_{1}\right)}^{2}}{{\left({\omega_{0}}^{2}-{\omega_{2}}^{2}\right)}^{2}+4{\beta_{\text{tot}}}^{2}{\omega_{2}}^{2}}\right]}^{1/2}$$

$$A_{3}=-\frac{P_{0}}{M\rho_{l}{R_{J}}^{2}}{\left[\frac{1+{\left(\omega_{3}R_{J}/c_{1}\right)}^{2}}{{\left({\omega_{0}}^{2}-{\omega_{3}}^{2}\right)}^{2}+4{\beta_{\text{tot}}}^{2}\omega_{3}^{2}}\right]}^{1/2}$$

$$K_{0}=\frac{C_{i}}{C_{0}}-\left(1+\frac{2\sigma }{P_{0}R_{J}}\right)$$

$$\begin{array}{c} K_{1}=\left[\left(1+\frac{2\sigma }{P_{0}R_{J}}\right)\left(4-3\kappa \right)-\frac{4C_{i}}{C_{0}}\right]\cdot \left\{-\frac{\omega_{1}^{2}}{4\omega_{0}^{2}M}+\frac{1}{4}\frac{1}{\rho_{l}R_{J}^{2}\omega_{0}^{2}M}\left[3\kappa \left(3\kappa +1\right)\left(P_{0}+\frac{2\sigma }{R_{J}}\right)-\frac{4\sigma }{R_{J}}\right]\right\}\\ +\left(1+\frac{2\sigma }{P_{0}R_{J}}\right)\frac{\left(4-3\kappa )(3-3\kappa \right)}{4}-\frac{3C_{i}}{C_{0}} \end{array}$$

$$\begin{array}{c} K_{2}=\left[\left(1+\frac{2\sigma }{P_{0}R_{J}}\right)\left(4-3\kappa \right)-\frac{4C_{i}}{C_{0}}\right]\cdot \left\{-\frac{\omega_{2}^{2}}{4\omega_{0}^{2}M}+\frac{1}{4}\frac{1}{\rho_{l}R_{J}^{2}\omega_{0}^{2}M}\left[3\kappa \left(3\kappa +1\right)\left(P_{0}+\frac{2\sigma }{R_{J}}\right)-\frac{4\sigma }{R_{J}}\right]\right\}\\ +\left(1+\frac{2\sigma }{P_{0}R_{J}}\right)\frac{\left(4-3\kappa )(3-3\kappa \right)}{4}-\frac{3C_{i}}{C_{0}} \end{array}$$

$$\begin{array}{c} K_{3}=\left[\left(1+\frac{2\sigma }{P_{0}R_{J}}\right)\left(4-3\kappa \right)-\frac{4C_{i}}{C_{0}}\right]\cdot \left\{-\frac{\omega_{3}^{2}}{4\omega_{0}^{2}M}+\frac{1}{4}\frac{1}{\rho_{l}R_{J}^{2}\omega_{0}^{2}M}\left[3\kappa \left(3\kappa +1\right)\left(P_{0}+\frac{2\sigma }{R_{J}}\right)-\frac{4\sigma }{R_{J}}\right]\right\}\\ +\left(1+\frac{2\sigma }{P_{0}R_{J}}\right)\frac{\left(4-3\kappa )(3-3\kappa \right)}{4}-\frac{3C_{i}}{C_{0}} \end{array}$$

(1) One crossing point on the right

We assume that the predicted threshold curves of the single-frequency approach with frequency $\omega_{1}$ and one of the triple frequencies intersect at the point $\left(R_{T1},P_{e,T1}\right)$. According to Eqs (5), (18), and (19), the point satisfies the following two equations, where $N_{1}{=N}_{2}$ change at the same time. In threshold curve of single frequency,

(A3) $$K_{0}=K_{1}\cdot A_{1}^{2}\cdot \left({P_{e,T1}}^{2}/{P_{0}}^{2}\right)$$

In the threshold curve of triple frequencies,

(A4) $$\begin{array}{c} K_{0}=K_{1}\cdot A_{1}^{2}\cdot \left({P_{TA1}}^{2}/P_{0}^{2}\right)+K_{2}\cdot {A_{2}}^{2}\cdot \left({P_{TA2}}^{2}/P_{0}^{2}\right)+K_{3}\cdot A_{3}^{2}\cdot \left({P_{AT3}}^{2}/P_{0}^{2}\right)\\ \left(=\left({P_{e,T1}}^{2}/P_{0}^{2}\right)\left(\frac{K_{1}\cdot A_{1}^{2}}{1+{N_{1}}^{2}+{N_{2}}^{2}}+\frac{K_{2}\cdot A_{2}^{2}\cdot {N_{1}}^{2}}{1+{N_{1}}^{2}+{N_{2}}^{2}}+\frac{K_{3}\cdot A_{3}^{2}\cdot {N_{2}}^{2}}{1+{N_{1}}^{2}+{N_{2}}^{2}}\right)\right] \end{array}$$

Using Eqs. (A3)–(A4), we get

(A5) $$2K_{1}\cdot A_{1}^{2}=K_{2}\cdot A_{2}^{2}+K_{3}\cdot A_{3}^{2}$$

Thus, $N_{1}$ and $N_{2}$ have no influence on the crossing point $\left(R_{T1},P_{e,T1}\right)$. Regardless of how the specific values of $N_{1}$ and $N_{2}$ change (as long as $N_{1}{=N}_{2}$), the predicted threshold curve of the single-frequency approach with frequency $\omega_{1}$ and all the predicted threshold curves of the triple frequencies intersect at this point.

(2) One crossing point on the left

Similarly, the predicted threshold curves of the single-frequency approach with frequency $\omega_{3}$ and one of the triple frequencies intersect at the point $\left(R_{T2},P_{e,T2}\right)$. The point satisfies the following two equations, where $N_{2}$ changes and $N_{1}$ remains constant. In the threshold curve of single frequency,

(A6) $$K_{0}=K_{3}\cdot {A_{3}}^{2}\cdot \left({P_{e,T2}}^{2}/{P_{0}}^{2}\right)$$

In the threshold curve of triple frequencies,

(A7) $$\begin{array}{c} K_{0}=K_{1}\cdot A_{1}^{2}\cdot \left({P_{TA1}}^{2}/P_{0}^{2}\right)+K_{2}\cdot A_{2}^{2}\cdot \left({P_{TA2}}^{2}/P_{0}^{2}\right)+K_{3}\cdot A_{3}^{2}\cdot \left({P_{AT3}}^{2}/P_{0}^{2}\right)\\ \left(=\left({P_{e,T2}}^{2}/P_{0}^{2}\right)\left(\frac{K_{1}\cdot A_{1}^{2}}{1+{N_{1}}^{2}+{N_{2}}^{2}}+\frac{K_{2}\cdot {A_{2}}^{2}\cdot N_{1}^{2}}{1+{N_{1}}^{2}+{N_{2}}^{2}}+\frac{K_{3}\cdot A_{3}^{2}\cdot N_{2}^{2}}{1+1+{N_{1}}^{2}+{N_{2}}^{2}}\right)\right] \end{array}$$

Using Eqs. (A6)–(A7),

(A8) $$\left(1+{N_{1}}^{2}\right)K_{3}\cdot {A_{3}}^{2}=K_{1}\cdot {A_{1}}^{2}+N_{1}\cdot K_{2}\cdot {A_{2}}^{2}$$

Thus, $N_{2}$ has no influence on the crossing point $\left(R_{T2},P_{e,T2}\right)$. Regardless of how the specific value of $N_{2}$ changes (as long as $N_{1}$ remains constant), the predicted threshold curves of the single-frequency approach with frequency $\omega_{3}$ and all the predicted threshold curves of the triple frequencies intersect at this point $\left(R_{T2},P_{e,T2}\right)$.

(3) Two crossing points

The predicted threshold curves of the single-frequency approach with frequency $\omega_{2}$ and one of the triple frequencies intersect at the points $\left(R_{T3},P_{e,T3}\right)$ and $\left(R_{T4},P_{e,T4}\right)$. The points satisfy the following two equations, where $N_{1}$ changes and $N_{2}$ remains constant. In the threshold curve of single frequency,

(A9) $$K_{0}=K_{2}\cdot {A_{2}}^{2}\cdot \left({P_{e,T3\;\text{or}\;4}}^{2}/{P_{0}}^{2}\right)$$

In threshold curve of triple frequencies,

(A10) $$\begin{array}{c} K_{0}=K_{1}\cdot A_{1}^{2}\cdot \left({P_{TA1}}^{2}/P_{0}^{2}\right)+K_{2}\cdot A_{2}^{2}\cdot \left({P_{TA2}}^{2}/P_{0}^{2}\right)+K_{3}\cdot A_{3}^{2}\cdot \left({P_{AT3}}^{2}/P_{0}^{2}\right)\\ \left(=\left({P_{e,T\;\text{or}\;4}}^{2}/P_{0}^{2}\right)\left(\frac{K_{1}\cdot {A_{1}}^{2}}{1+{N_{1}}^{2}+{N_{2}}^{2}}+\frac{K_{2}\cdot {A_{2}}^{2}\cdot {N_{1}}^{2}}{1+{N_{1}}^{2}+{N_{2}}^{2}}+\frac{K_{3}\cdot {A_{3}}^{2}\cdot {N_{2}}^{2}}{1+{N_{1}}^{2}+{N_{2}}^{2}}\right)\right] \end{array}$$

Using Eqs. (A9) and (A10),

(A11) $$\left(1+{N_{2}}^{2}\right)K_{2}\cdot {A_{2}}^{2}=K_{1}\cdot {A_{1}}^{2}+N_{2}\cdot K_{3}\cdot {A_{3}}^{2}$$

Thus, $N_{1}$ has no influence on the two crossing points. Regardless of the value of $N_{1}$ changes (as long as $N_{2}$ remains constant), the predicted threshold curves of the single-frequency approach with frequency $\omega_{2}$ and all the predicted threshold curves of the triple frequencies intersect at the two points $\left(R_{T3},P_{e,T3}\right)$ and $\left(R_{T4},P_{e,T4}\right)$.
